# Predictive value of machine learning algorithm of coronary artery calcium score and clinical factors for obstructive coronary artery disease in hypertensive patients

**DOI:** 10.1186/s12911-023-02352-8

**Published:** 2023-10-30

**Authors:** Minxian Wang, Mengting Sun, Yao Yu, Xinsheng Li, Yongkui Ren, Da Yin

**Affiliations:** 1 Department of Cardiology, the First Affiliated Hospital of Dalian Medical University, No. 222 Zhongshan Road, Zhongshan District, Dalian, Liaoning Province China; 2 Department of Cardiology, Shenzhen People’s Hospital, 2nd clinical medical college of JINAN university, 1st affiliated hospital of the southern university of Science and Technology, No. 1017 Dongmen North Road, Luohu District, Shenzhen, Guangdong Province China

**Keywords:** Obstructive coronary artery Disease, Machine learning, Coronary artery calcium, Hypertension.

## Abstract

**Background:**

The addition of coronary artery calcium score (CACS) to prediction models has been verified to improve performance. Machine learning (ML) algorithms become important medical tools in an era of precision medicine, However, combined utility by CACS and ML algorithms in hypertensive patients to forecast obstructive coronary artery disease (CAD) on coronary computed tomography angiography (CCTA) is rare.

**Methods:**

This retrospective study was composed of 1,273 individuals with hypertension and without a history of CAD, who underwent dual-source computed tomography evaluation. We applied five ML algorithms, coupled with clinical factors, imaging parameters, and CACS to construct predictive models. Moreover, 80% individuals were randomly taken as a training set on which 5-fold cross-validation was done and the remaining 20% were regarded as a validation set.

**Results:**

16.7% (212 out of 1,273) of hypertensive patients had obstructive CAD. Extreme Gradient Boosting (XGBoost) posted the biggest area under the receiver operator characteristic curve (AUC) of 0.83 in five ML algorithms. Continuous net reclassification improvement (NRI) was 0.55 (95% CI (0.39–0.71), *p* < 0.001), and integrated discrimination improvement (IDI) was 0.04 (95% CI (0.01–0. 07), *p* = 0.0048) when the XGBoost model was compared with traditional Models. In the subgroup analysis stratified by hypertension levels, XGBoost still had excellent performance.

**Conclusion:**

The ML model incorporating clinical features and CACS may accurately forecast the presence of obstructive CAD on CCTA among hypertensive patients. XGBoost is superior to other ML algorithms.

**Supplementary Information:**

The online version contains supplementary material available at 10.1186/s12911-023-02352-8.

## Introduction

Hypertension affects approximately one-third of the world’s adult population and is a major risk for the presence of coronary artery disease (CAD) [1]. Arguably the biggest challenge for cardiologists is to more accurately identify patients with obstructive CAD among all individuals with hypertension. Coincidentally, coronary computed tomography angiography (CCTA) has emerged as a non-invasive and popular method for the evaluation of CAD for many years [2, 3]. With the extensive application of CCTA in clinical practice, it is imperative to optimize patient selection to improve diagnostic yield and cost-effectiveness of CCTA [4].

The coronary artery calcium (CAC) scan, different from the CCTA, can be accomplished with 10 to 15 min of total room time at about 1 mSy of radiation, without the need for contrast agents [5]. CAC, as a biomarker of subclinical atherosclerosis, is the most significant independent predictor for cardiovascular events as well as all-cause mortality [6, 7]. Furthermore, accumulated evidence has demonstrated that the absence of coronary artery calcium (CAC) in CCTA represents a low risk for the incidence of cardiovascular events while there is increased cardiovascular risk with CACS increasing [8, 9]. In addition, the addition of CACS to clinical prediction models has been revealed to improve predictive performance for CAD [10, 11]. Interestingly, previous reports have unveiled that CAC is not only accelerated by hypertension but also contributes to hypertension [12]. However, the predictive importance of CACS for obstructive CAD in hypertensive patients has rarely been defined.

Machine learning (ML) is an emerging sort of artificial intelligence (AI) and is skilled at uniting diverse population characteristics to fit superior prediction models. Thus, ML has been widely applied to healthcare data analysis in recent years [13, 14]. By taking full advantage of the powerful prediction ability of ML algorithms, it may be feasible to develop prediction tools that surpass traditional statistical models in some cases, thus optimizing the prediction of CAD and decreasing the extensive use of CCTA in hypertensive patients. In this study, we seek to develop ML-based models integrating clinical factors and CACS, to forecast the presence of obstructive CAD on CCTA among patients with hypertension.

## Methods

### Study population and definition

We retrospectively screened 1,346 hypertensive patients without a history of CAD who were admitted to the Department of Hypertension and underwent CCTA examination in the First Affiliated Hospital of Dalian Medical University from January 2014 to December 2017. Hypertension is defined as a prior diagnosis of hypertension or the use of antihypertensive medications. Definition of hypertension is based on the 2017 ACC/AHA guideline (systolic blood pressure (SBP) ≥ 140 mm Hg and/or diastolic blood pressure (DBP) ≥ 90 mm Hg). Meanwhile, patients previously diagnosed with CAD according to CCTA, coronary angiography, treadmill exercise testing, and (or) typical chest pain symptoms were excluded. Additional exclusion criteria were missing data of scan identifiers, uncertain date of birth, and unavailable CACS. Patients with severe hepatic/renal insufficiency, malignant disease, and poor CCTA image quality were excluded. Moreover, laboratory parameters were from fasting venous blood which was collected on the second morning of admission and detected in the biochemistry lab of the First Affiliated Hospital of Dalian Medical University.

**Fig. 1:**
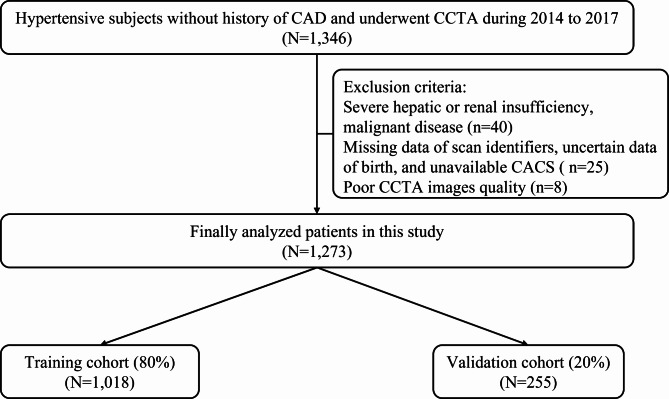
Flow chart of the study CAD, coronary artery disease; CCTA, coronary computed tomography angiography; CACS, coronary artery calcium score

The data included baseline patient characteristics, the results of blood tests, and imaging data in the preliminary experiment. For data preprocessing, we removed variables that have no clinical significance, and deleted some variables that have no obvious causal relationship with the outcomes. The dataset was imputed using multiple imputation. Then, the recursive feature elimination (RFE) algorithm was used to select key variables and develop machine learning model. Finally, A total of 68 variables from 1,273 people were eventually applied in the study (Detailed study flow was shown in Fig. 1, and the list of included variables is shown in Supplementary Table 1). Furthermore, all the individuals were randomly distributed into two sets, namely the training set (80%) for ML model development and the validation set (20%) for performance evaluation. Furthermore, random splitting was repeated until the patients were equally distributed in both sets. The comparable differences in baseline characteristics between the training set and the validation set were shown in Supplementary Table 2.

### Coronary computed tomography angiography and coronary artery calcium scanning

According to the guidelines outlined by the Society of Cardiovascular Computed Tomography, CCTA image acquisition, and processing, as well as coronary artery calcium scanning, were performed on the scanner (dual-source, Somatom Definition CT, Siemens, Erlangen, Germany). Two professional imaging physicians blind to the patients’ clinical data independently evaluated all images to determine the extent of CAD and provide a CACS using the Agatston method which semi-automatically calculates a weighted sum of the area of coronary calcification in line with the available study [15]. The presence of diameter stenosis ≥ 50% in any of the four major epicardial coronary arteries detected on CCTA was defined as obstructive CAD.

### The optimal machine learning model

Five types of ML algorithms were performed to model our data: Extreme Gradient Boosting (XGBoost), Random Forest (RF), Support Vector Machine (SVM), Neural Network (NNET), and traditional Logistic Regression (LR). The traditional LR model used in this study was composed of traditional clinical factors (including age, gender, stage of hypertension, history of diabetes mellitus, current smoker, as well as the levels of total cholesterol and triglyceride) and CACS. Moreover, the remaining 4 models included all available variables. Meanwhile, tuning was considered to avoid overfitting for ML-based models and the optimal hyper-parameter in the training process for ML models was 5-fold cross-validation. Followingly, the R software was applied to further train the ML algorithms to forecast the presence of obstructive CAD. Furthermore, the best-performing algorithm (with the highest area under the receiver operator characteristic curve (AUC)) was used to construct the classifier from the whole training set, with the same hyper-parameter, and applied it to the validation set to independently assess the predictive performance [16]. Additionally, shapely additive explanations (SHAP) was calculated to assess the feature ranking, as previously described [17].

### Statistical analysis

Kolmogorov–Smirnov test was applied to assess the distribution of continuous variables. Normally distributed continuous variables were expressed as mean (standard deviation (SD)) and compared with the t-test; non-normally distributed continuous variables were expressed as median (interquartile range) and compared with the non-parametric test. Fisher’s exact test was used to assess the differences between categorical variables, which are reported as a number (percentage). A multivariable logistic regression analysis with backward stepwise selection was applied to verify the independent risk factors of obstructive CAD and related results were reported as odds ratios (ORs) (95% confidence intervals (CIs)).

Five ML algorithms were compared to find the best algorithm. Further, the optimal algorithm was compared with the traditional LR regression using the calibration curve and Hosmer-Lemeshow test. In the subgroup analysis of hypertension, the corresponding sensitivity, specificity, positive predictive value, negative predictive value, as well as overall accuracy of ML algorithms were calculated. In addition, net reclassification improvement (NRI) and integrated discrimination improvement (IDI) were applied to compare predictive performance between the best ML algorithm and the traditional LR model. Additionally, SHAP was calculated to assess the importance of variables included in the XGBoost model. R software (https://www.r-project.org/) was used in statistical analyses. A two-tailed *p* < 0.05 was considered statistically significant.

## Results

### Demographic features

A total of 1,273 patients were finally included in the study and divided into two groups according to the presence of obstructive CAD (shown in Table 1). The prevalence of obstructive CAD was 16.3% (212 out of 1,273). Moreover, the proportion of CACS > 0 in the general population was 54.6% (695 out of 1,273), and 92% (195 out of 212) in obstructive CAD. The prevalence of males, previous diabetes mellitus, and current smokers was significantly higher in the obstructive CAD group (*p* < 0.05). The differences in CACS between the two groups were obvious (*p* < 0.05).

**Table 1:** Baseline characteristics of the study population

		Total (n = 1,273)	NO-OCAD (n = 1,061)	OCAD (n = 212)	KS Test *p*	*p*	
Age, y		58.2(14.3)	56.8(14.2)	65.2(12.8)	0.128	0.047	
Male, n (%)		692(54.4)	558(52.6)	134(63.2)		0.005	
Hypertension, n (%)						0.074	
Stage 1		55(4.3)	52(4.9)	3(1.4)			
Stage 2		107(8.4)	89(8.4)	18(8.5)			
Stage 3		1,111(87.3)	920(86.7)	191(90.1)			
Diabetes mellitus, n (%)		337(26.5)	255(24.0)	82(38.7)		0.000	
Current smoker, n (%)		362(28.4)	280(26.4)	82(38.7)		0.000	
Hs-CRP		1.0(0.5–2.3)	1.0(0.5–2.2)	1.1(0.5–2.8)	0.000	0.134	
eGFR (mL/min·1.73m^2^)		88.6(24.7)	97.7(28.0)	84.3(25.4)	0.568	0.144	
LVEF, %		59.0(58.0–59.0)	59.0(58.0–59.0)	59.0(58.0–59.0)	0.000	0.022	
TC, mmol/L		4.8(4.1–5.5)	4.8(4.5–5.5)	4.8(4.0-5.5)	0.009	0.652	
TG, mmol/L		1.4(1.0-2.1)	1.4(1.0–2.0)	1.6(1.1–2.2)	0.000	0.043	
HDL-C, mmol/L		1.2(0.9–1.4)	1.2(0.9–1.4)	1.1(0.9–1.3)	0.000	0.016	
LDL-C, mmol/L		2.6(2.2–3.1)	2.6(2.2–3.1)	2.6(2.1–3.2)	0.006	0.814	
CACS > 0, n (%)		695(54.6)	500(47.1)	195(92.0)		0.000	
CACS, score		2.2(0.0-116.0)	0.0(0.0-43.5)	292.0(58.3–745.0)	0.000	0.000	

Values are presented as mean (SD), median (25th–75th percentiles) or n (%).

OCAD, obstructive coronary artery disease; TC, total cholesterol; TG, triglyceride; HDL-C, high density lipoprotein cholesterol; LDL-C, low density lipoprotein cholesterol; eGFR, estimated glomerular filtration rate; hs-CRP, high-sensitivity C reactive protein; CACS, coronary artery calcium score. KS Test, Kolmogorov Smirnov Test.

### Univariate and multivariate logistic regression analysis of obstructive CAD

In univariate analysis, age, gender, history of diabetes mellitus, current smoker, and CACS were all significantly linked to obstructive CAD (*p* < 0.05), whereas there was no significant difference in total cholesterol levels. In multivariable logistic regression analysis, the results revealed that age (OR 1.035, 95% CI (1.021–1.050), *p* < 0.001), current smoker (OR 1.699, 95% CI (1.108–2.626), *p* = 0.016), and CACS (OR1.002, 95% CI (1.001–1.002), *p* < 0.001) were independently related to obstructive CAD. In addition, hypertension [stage 2 vs. stage 1 (OR 3.433, 95% CI (1.099–12.82), *p* = 0.046); stage 3 vs. stage 1(OR 3.373, 95% CI (1.254–10.932), *p* = 0.030)] was a positive predictor for obstructive CAD (shown in Table 2).

**Table 2:** Univariate and multivariate logistic regression analysis for obstructive CAD

Variables	Univariate analysis		Multivariate analysis	
	OR (95%CI)	*p*	OR (95%CI)	*p*
Male	1.549(1.143–2.099)	0.005	1.385(0.907–2.104)	0.128
Age	1.046(1.034–1.058)	0.000	1.035(1.021–1.050)	0.000
Hypertension				
Stage1	-[Reference]		- [Reference]	
Stage2	3.506(0.985–12.473)	0.053	3.433(1.099–12.82)	0.046
Stage 3	3.599(1.112–11.643)	0.033	3.272(1.254–10.932)	0.030
Diabetes mellitus	1.994(1.462–2.719)	0.000	1.252(0.874–1.779)	0.215
Current smoker	1.759(1.293–2.395)	0.000	1.699(1.108–2.626)	0.016
TC	0.995(0.867–1.152)	0.995		
CACS	1.002(1.002–1.003)	0.000	1.002(1.001–1.002)	0.000

TC, total cholesterol; CACS, coronary artery calcium score.

In the multivariate analysis, male, age, hypertension, diabetes mellitus, current smoker and CACS were adjusted.

### Performance of machine learning algorithm for obstructive CAD

**Fig. 2:**
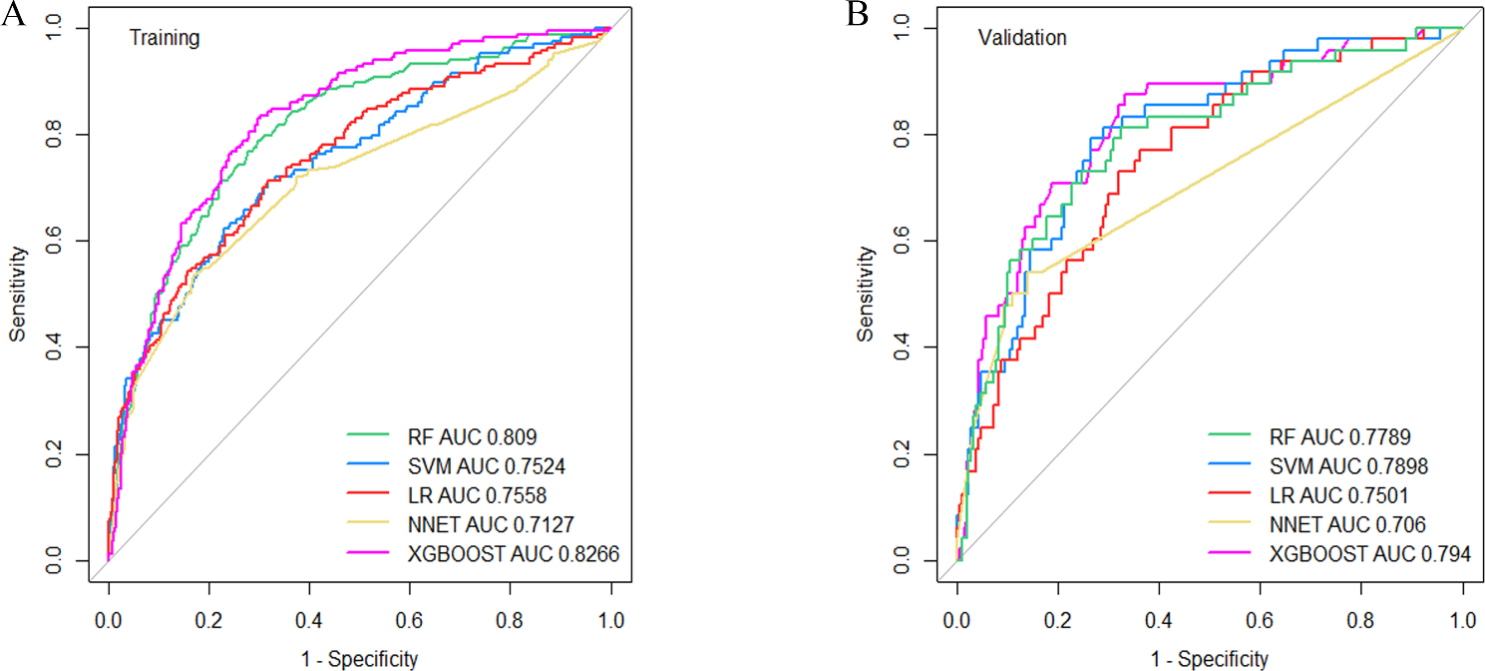
The area under the curve as a measure of individual model performance for the prediction of obstructive coronary artery disease on coronary computed tomography angiography in training cohort (**A**) and validation cohort (**B**) AUC, area under the curve; LR, Logistic Regression; XGBoost, Extreme Gradient Boosting; RF, Random Forest; SVM, Support Vector Machine; NNET, Neural Network

**Fig. 3:**
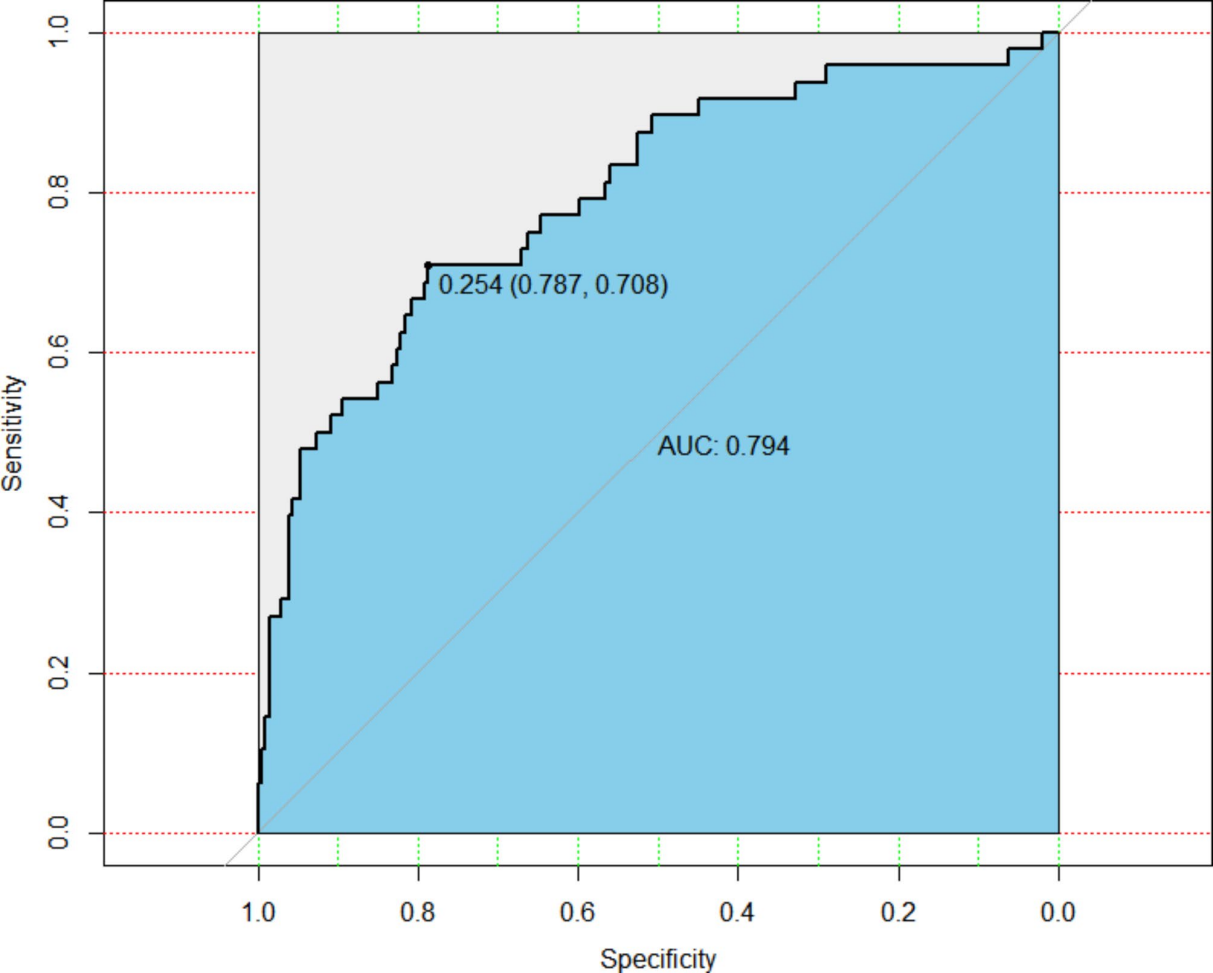
The receiver operating characteristic curve from applying the best-performing classifier (XGBoost) built in validation cohort AUC, the area under the curve; XGBoost, Extreme Gradient Boosting

Within the training cohort and validation cohort, comparisons of the performance of the five ML algorithms models were detailed in Fig. 2, and their performance was evaluated based on the area under the receiver operating characteristics curve (AUC) through 5-fold cross-validation (AUC of the RF model (SD) = 0.8090(0.04); AUC of the SVM model (SD) = 0.7524 (0.05); AUC of the LR model (SD) = 0.7558 (0.03); AUC of the XGBoost model (SD) = 0.8266 (0.03); AUC of the NNET (SD) = 0.7127(0.07)). The predictive value and optimal cutoff in the different machine learning algorithms for obstructive CAD were presented in Table 3. Moreover, XGBoost, as the best-performing algorithm, achieved a high AUC of 0.794 in the independent validation set (shown in Fig. 3).

**Table 3:** The predictive value and optimal cutoff in the different models

	Cutpoint	Sensitivity	Specificity	PPV	NPV	accuracy
LR	0.121	0.726	0.702	0.319	0.930	0.706
RF	0.184	0.768	0.722	0.347	0.942	0.730
SVM	0.134	0.713	0.681	0.301	0.925	0.687
NNET	0.323	0.511	0.726	0.447	0.875	0.618
XGBoost	0.254	0.837	0.786	0.389	0.950	0.767

PPV, positive predict value; NPV, negative predict value. LR, Logistic Regression; XGBoost, Extreme Gradient Boosting; RF, Random Forest; SVM, Support Vector Machine; NNET, Neural Network; PPV, positive predictive value; NPV, negative predictive value

**Fig. 4:**
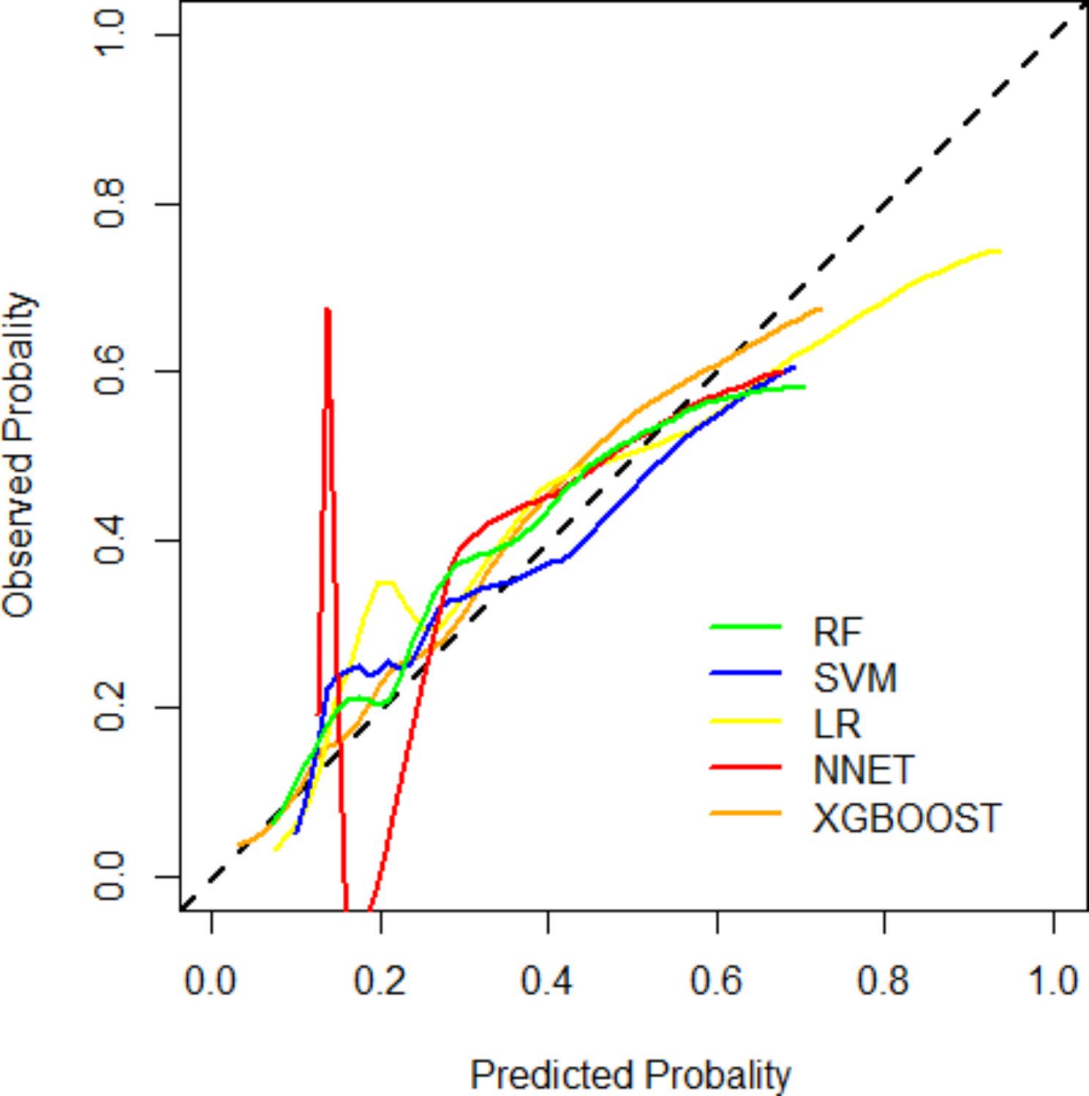
Calibration curve for different models for prediction of the likelihood of obstructive CAD LR, Logistic Regression; XGBoost, Extreme Gradient Boosting; RF, Random Forest; SVM, Support Vector Machine; NNET, Neural Network; CAD, coronary artery disease

To evaluate the deterministic of a given new observation belonging to one of the already established sorts (prediction value for the presence or absence of CAD on CCTA), Model calibration was applied (shown in Fig. 4). Interestingly, the minimum difference between the predicted and observed likelihood of obstructive CAD appeared in the XGBoost model. That is, the XGBoost model achieved a good model fit. Further, the Hosmer-Lemeshow test indicates that the XGBoost model had a high calibration (*p* = 0.301), while the traditional LR model was disappointing (*p* < 0.05). Additionally, continuous NRI was 0.55 (95% CI (0.39–0.71), *p* < 0.001), IDI was 0.04 (95% CI (0.01–0. 07), *p* = 0.0048) when the XGBoost model was compared with LR Models.

### Feature importance in the XGBoost model

**Fig. 5:**
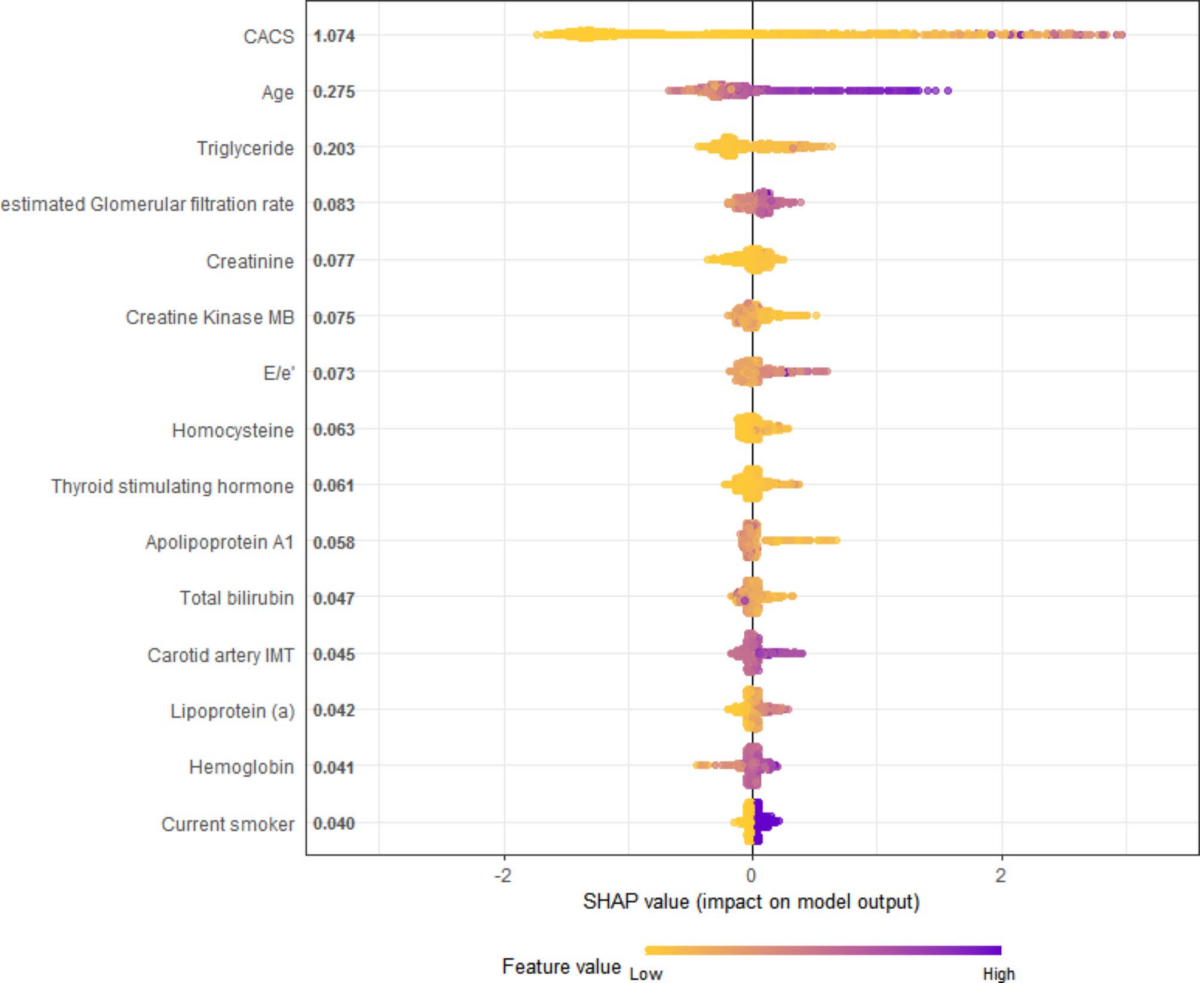
Feature importance plot in the XGBoost model The top 15 clinical variables are shown. The yellow and purple points in each row represent participants having low to high values of the specific variable, while the x-axis gives the SHAP value which affects the model [i.e. does it tend to drive the predictions towards the event (positive value of SHAP) or non-event (negative value of SHAP)] CACS, coronary artery calcium score; XGBoost, extreme gradient boosting; SHAP, Shapley additive explanation values; E/e’, early diastolic transmitral velocity to early mitral annulus diastolic velocity ratio; Carotid artery IMT, Carotid artery intima-media thickness

As shown in Fig. 5, the probability of the prevalence of obstructive CAD increased, with CACS increasing. That is, CACS had the highest predictive value for the presence of obstructive CAD. Age was the second important variable and was followed by plasma triglycerides levels, estimated glomerular filtration rate (eGFR), and plasma creatinine levels. Interestingly, carotid intima-media thickness was also related to obstructive CAD among imaging parameters.

### Subgroup analysis stratified by Hypertension levels

Because the proportion of patients in stage 1 and stage 2 hypertension was low, we grouped patients according to whether they were in stage 3 hypertension or not. The proportion of CAC > 0 in stage 3 hypertension was significantly higher than that in patients without stage 3 hypertension, and a similar result was found with CACS as a continuous variable(*p* < 0.05) (shown in Table 4). The sensitivity, specificity, positive predictive value, negative predictive value, and accuracy of the XGBoost model for obstructive CAD in patients with stage 3 hypertension were 82.0%, 87.6%, 57.9%, 93.1%, and 84.8%, respectively; results were and 86.9%, 90.5%, 53.3%, 92.3%, and 88.7% in non-stage 3 hypertensive patients (shown in Table 5).

**Table 4:** Baseline characteristics in different hypertensive subgroups

	Hypertension (stage3)	Hypertension (stage1/2)	*p*
Age, y	58.8(14.2)	54.9(14.7)	0.001
Male, n (%)	592(54.2)	100(55.2)	0.795
Diabetes, n (%)	310(28.4%)	27(14.9%)	0.000
eGFR, (mL/min·1.73m^2^)	94.7(26.9)	100.9(29.1)	0.005
Cre, mmol/L	66.0(54.0-76.4)	65.0(52.0-76.2)	0.220
TC, mmol/L	4.8(1.1)	4.7(1.0)	0.532
TG, mmol/L	1.5(1.0-2.1)	1.3(1.0-1.9)	0.097
LDL-C, mmol/L	2.6(0.7)	2.6(0.6)	0.570
CACS > 0, n (%)	617(56.5)	78(43.1)	0.028
CACS, score	4.7(0.0-139.0)	0.0(0.0–56.0)	0.003

Values are presented as mean (SD), median (25th–75th percentiles) or n (%)

CACS, coronary artery calcium score; TC, total cholesterol; TG, triglyceride; HDL-C, high density lipoprotein cholesterol; LDL-C, low density lipoprotein cholesterol; eGFR, estimated glomerular filtration rate; Cre, creatinine

**Table 5:** The predictive value and optimal cutoff stratified by subgroups in XGBoost

	Cutpoint	Sensitivity	Specificity	PPV	NPV	accuracy
XGBoost	0.254	0.837	0.786	0.389	0.950	0.767
Stage 3	0.224	0.820	0.876	0.579	0.931	0.848
Stage 1/2	0.202	0.869	0.905	0.533	0.923	0.887

PPV, positive predict value; NPV, negative predict value. XGBoost, Extreme Gradient Boosting; PPV, positive predictive value; NPV, negative predictive value.

## Discussion

In this study, we developed and validated multiple popular ML algorithms to forecast the presence of obstructive CAD in hypertensive patients. A comparison among five ML algorithms demonstrated that the XGBoost model was the most excellent in terms of predictive power and appropriate for patients with different levels of blood pressure (BP). The ML algorithm-based model was potentially able to guide clinical decision-making and improve risk stratification in hypertensive patients. In addition, this study further emphasized the importance of CACS as a risk stratification tool in hypertensive patients.

### The importance of machine learning

The current study demonstrated that ML algorithms are necessary and applicable in the context of clinical requirements. Furthermore, the XGBoost model is the most appropriate model among the five ML algorithms in terms of predictive power for the presence of obstructive CAD in hypertensive patients and is superior to traditional regression models. CAD is a common and frequently-occurring disease linked to high morbidity, mortality, and healthcare expenditure. To invasively forecast the occurrence of CAD, many models have been developed. Nevertheless, the performance of most of the existing models is limited to the presence of CAD [18–20]. Additionally, the discriminative ability of several models becomes lower, when they have been validated in more than one external population [21]. This downward trend may be partly attributed to the utilization of diverse imaging modalities as well as the different definition of obstructive CAD, and model complexity. Importantly, with the development of social and extensive popularization of health knowledge, dietary habits, environmental exposures, and preventative practices are ever-changing. Therefore, previous models may not be comprehensive. That is, there is an urgent need for optimal predictive models for obstructive CAD in hypertensive patients. ML algorithms became an available and suitable option, as a result of two inherent characteristics. On the one hand, ML algorithms are superior to the one-dimensional traditional statistical methods in terms of finding correlations between variables; on the other hand, ML algorithms are optimal to make use of increasingly complex data that is pivotal to improving prediction performance. And not coincidentally, ML algorithms have been verified to be a powerful predictive tool in the context of cardiovascular applications [22–24]. Similarly, the predictive power of ML for obstructive CAD was superior to traditional models in this study. Meanwhile, the XGBoost model may be the optimal model given calibration and predictive performance for the presence of obstructive CAD in patients with hypertension. This study upholds that intermediate to high-risk hypertensive patients evaluated by the XGBoost model to directly receive further testing such as CCTA and coronary angiography, as well as preventive therapies, may be reasonable and cost-efficient.

### The importance of coronary artery calcium score

The current study uncovered that CACS is the most important factor among the diverse clinical parameters that can stratify hypertensive patients with the risk of obstructive CAD. Previous researchers have unveiled that the performance of predictive models was markedly improved by the addition of CACS [10, 11, 25]. For example, the C-statistic increased from 0.79 to 0.88 with the addition of the CACS to extend CAD clinical score to forecast the presence of obstructive CAD on invasive coronary angiography [26]. Furthermore, the Heinz Nixdorf Recall (HNR) study demonstrated the absence of coronary calcium represents a relatively low CVD risk regardless of BP stage in hypertensive patients. Namely, CAC was a more robust predictor for cardiovascular events than BP levels in the HNR study. Whatever in any BP category, the adjusted hazard ratios of cardiovascular events grew with the increase of CACS. Meanwhile, an increasing BP level played no (or only a modest) role in CAD risk within each CACS category [27]. Taken together, the accumulated studies support that CACS is very valuable to optimize risk stratification in hypertensive patients. Most previous researches prospectively focused on the relations between CACS and MACEs, however, cross-sectional studies evaluating the predictive performance of CACS for obstructive CAD in patients with hypertension were rare. Our study from cross-sectional data unveiled that CACS is a superior predictor for the occurrence of obstructive CAD in patients with different blood pressure levels. Given its high predictive value, CACS may be an applicable tool to guide clinical decision-making and optimize treatment strategies even in patients with prehypertension and mild hypertension, while without the symptoms of CAD. Our investigation greatly enhances the evidence of CACS as a significant risk stratification tool in hypertensive adults and supports a stronger recommendation of the CACS in future clinical guidelines.

### The relationship between Hypertension and coronary artery calcification

To a certain extent, this study further verified that hypertension and coronary artery calcification mutually reinforce. With the aging population and epidemic of obesity in recent years, the prevalence of hypertension is scheduled to gradually increase in the future. Moreover, hypertension is an independent risk for the development of atherosclerosis [28]. Therefore, rational methods about how to early and non-invasively forecast the prevalence of CAD attract more and more attention in patients with hypertension. As far as we know, vascular calcifications are not only accelerated by hypertension but also contribute to hypertension. Current consensus holds that vascular calcification, either intimal or medial, may directly increase arterial stiffness. Alternatively, arterial stiffness is closely linked to raised blood pressure [29]. Parallelly, the proportion of patients with CACS > 0 and CACS was significantly higher in the stage 3 hypertension group than in other groups in this study. Similar results were seen in the proportion of patients with diabetes, possibly because there is a large overlap in etiology between hypertension and diabetes, the level of hypertension is closely related to the proportion of patients with diabetes [30]. Additionally, consistent with previous reports [10, 17], CACS had a higher negative value for obstructive CAD. The reasonable explanation is that the presence of calcification may affect the accuracy of CCTA in assessing the degree of coronary artery stenosis.

### Limitation

Several limitations of the present study should be paid more attention to. Firstly, the present investigation was lack of external validation in an independent cohort, which was planned for subsequent analysis. Secondly, the presence of severe calcification may lead to overestimates stenosis on CCTA. Hence, more than 50% stenosis on CCTA may not represent the accuracy > 50% stenosis evaluated by coronary angiography. Finally, the number of patients with stage 1 and stage 2 hypertension in our cohort was too small to be stratified separately, we will expand the sample size for further research.

## Conclusions

The ML model, especially The XGBoost model, incorporating clinical features and CACS may accurately forecast the presence of obstructive CAD on CCTA among patients with hypertension. It may be reasonable for intermediate to high-risk hypertensive patients evaluated by the XGBoost model to directly receive further testing such as CCTA and coronary angiography, as well as preventive therapies.

### Electronic supplementary material

Below is the link to the electronic supplementary material.

**Supplementary Table 1** Pre-implant clinical features included in the analysis. **Supplementary Table 2** Baseline characteristics for training and validation sets

## Data Availability

The data are available from the corresponding authors upon reasonable request.
