# Supplementary material for: Predictive value of machine learning algorithm of coronary artery calcium score and clinical factors for obstructive coronary artery disease in hypertensive patients
Source: BMC Med Inform Decis Mak. 2023 Oct 30;23:244. doi: 10.1186/s12911-023-02352-8 (PMC10617081; doi:10.1186/s12911-023-02352-8)
Supplement: Supplementary file 1 — Supplementary Table 1 Pre-implant clinical features included in the analysis. Supplementary Table 2 Baseline characteristics for training and validation sets [file 12911_2023_2352_MOESM1_ESM.docx]

**Supplementary Table 1** Pre-implant clinical features included in the analysis

| **Demographics** | **Comorbidities** | **ECG/** **imaging parameters** | **Laboratory Results** | |
| --- | --- | --- | --- | --- |
| Age  Gender  Height  Weight  SBP | Stage1  Stage2  Stage3  Diabetes  Stroke  Current smoker  Atrial fibrillation  Tumour  OSA  PAD | Heart rate  P-R interval.  QRS duration  QT Interval  Corrected QT Interval  Left atrial diameter  Right atrial diameter  Right ventricular diameter  Left Ventricular Hypertrophy  LVEF  [LVDD](http://abbr.dict.cn/left%20ventricular%20diastolic%20dimension/lvdd)  IVST  E/A ratio  E/e'  R-Carotid-IMT  L-Carotid-IMT  CACS | White blood cell  Haemoglobin  Platelets  Neutrophils  hs-CRP  NT-proBNP  D-dimer  Creatine kinase  Isoenzyme  Troponin I  Total cholesterol  Triglycerides  HDL-C  LDL-C  Alkaline Phosphatase  AST  Total bilirubin  Alanine Aminotransferase | γ-Glutamyltransferase  Homocysteine  Lipoprotein a  Apolipoprotein AI  Apolipoprotein B  Glucose  Hemoglobin A1c  [eGFR](http://abbr.dict.cn/Estimated%20Glomerular%20Filtration%20Rate/EGFR)  Serum uric acid  Serum urea  TSH  Free Triiodothyronine  Free thyroxine  Serum sodium  Serum potassium  Serum calcium |
| SBP, systolic blood pressure; OSA, Obstructive Sleep Apnea; LVEF, left ventricular ejection fraction; LVDD, left ventricular end diastolic dimension; IVST, interventricular septal thickness; E/A ratio, the ratio of E to mitral peak velocity of late filling; E/e', the ratio of mitral peak velocity of early filling to early diastolic mitral annular velocity;IMT, intima-media thickness; CACS, Coronary artery calcium score; hs-CRP, high-sensitivity C-reactive protein; HDL-C, high-density lipoprotein cholesterol; LDL-C, low-density lipoprotein cholesterol; TSH, thyroid-stimulating hormone; eGFR, Estimated Glomerular Filtration Rate; AST, Aspartate Aminotransferase. | | | | |

**Supplementary Table 2** Baseline characteristics for training and validation sets

|  | [ALL] | Test Cohort | Training Cohort | *p*.overall |
| --- | --- | --- | --- | --- |
|  | N=1273 | N=255 | N=1018 |  |
| Age, y | 58.2 (14.4) | 59.0 (13.8) | 58.0 (14.5) | 0.285 |
| Male, n (%) | 692 (54.4%) | 149 (58.4%) | 543 (53.3%) | 0.165 |
| Diabetes mellitus, n (%) | 337 (26.5%) | 74 (29.0%) | 263 (25.8%) | 0.341 |
| Current smoker, n (%) | 362 (28.4%) | 82 (32.2%) | 280 (27.5%) | 0.163 |
| TC, mmol/L | 4.83 (1.05) | 4.75 (1.04) | 4.85 (1.05) | 0.174 |
| TG, mmol/L | 1.45 [1.04-2.04] | 1.37 [1.02-2.05] | 1.46 [1.05-2.04] | 0.501 |
| HDL-C, mmol/L | 1.15 [0.98-1.35] | 1.15 [0.98-1.37] | 1.15 [0.98-1.34] | 0.744 |
| LDL-C, mmol/L | 2.64 (0.71) | 2.59 (0.69) | 2.66 (0.72) | 0.159 |
| eGFR(mL/min·1.73m^2^) | 66.0 [54.0-76.4] | 66.0 [54.0-77.0] | 65.0 [54.0-76.4] | 0.715 |
| LVEF, % | 59.0 [58.0-59.0] | 59.0 [58.0-59.0] | 59.0 [58.0-59.0] | 0.877 |
| OCAD, n (%) | 212 (16.7%) | 48 (18.8%) | 164 (16.1%) | 0.344 |
| CACS, AU | 2.20 [0.00-116] | 11.1 [0.00-162] | 0.95 [0.00-108] | 0.087 |
| CACS |  |  |  | 0.149 |
| 0 | 578 (45.4%) | 103 (40.4%) | 475 (46.7%) |  |
| 1-99 | 363 (28.5%) | 80 (31.4%) | 283 (27.8%) |  |
| 100-399 | 188 (14.8%) | 46 (18.0%) | 142 (13.9%) |  |
| ≥400 | 144 (11.3%) | 26 (10.2%) | 118 (11.6%) |  |

Values are presented as mean (SD), median (25th–75th percentiles) or n (%). TC, total cholesterol; TG, triglyceride; HDL-C, high density lipoprotein cholesterol; LDL-C, low density lipoprotein cholesterol; eGFR, estimated glomerular filtration rate; hs-CRP, high-sensitivity C reactive protein; CACS, coronary artery calcium score.
